# The Electric–Thermal Effect of a Carbon-Fibre-Reinforced Epoxy Composite and Its Corresponding Mechanical Properties

**DOI:** 10.3390/polym14214489

**Published:** 2022-10-24

**Authors:** Runtian Zhu, Guoxian Wang, Yuebin Lin, Jinxi Long, Longji Du, Xusheng Du, Rajab Abousnina, T. Tafsirojjaman

**Affiliations:** 1 Key Laboratory for Transport Industry of Bridge Detection Reinforcement Technology, Chang’an University, Xi’an 710064, China; 2 Zhuhai Communication Group, Zhuhai 519000, China; 3 The Key Laboratory of Urban Security and Disaster Engineering of Ministry of Education, Beijing University of Technology, Pingleyuan Road 100, Beijing 100124, China; 4 Highway Bridges National Engineering Research Center, Beijing 100088, China; 5 Institute of Advanced Wear & Corrosion Resistant and Functional Materials, Jinan University, Guangzhou 510632, China; 6 School of Civil and Mechanical Engineering, Curtin University, Perth 6102, Australia; 7 School of Civil, Environmental and Mining Engineering, The University of Adelaide, Adelaide 5005, Australia

**Keywords:** composites, electric–thermal response, epoxy resin, flexural strength, interfacial property

## Abstract

In this work, the electric–thermal effect of a carbon-fibre-reinforced epoxy composite (CFRE) panel was studied, as well as the influence of the electric heating treatment on the mechanical properties of the composite. It was observed that the temperature of the composite increased rapidly once the current was loaded, and the equilibrium surface temperature was reached within 2 min. The electric–thermal effect and mechanical properties depended on both the current loading time and the current intensity. At 5A, the flexural modulus and strength of the CFRE increased before decreasing with the current loading time. Under the same treatment time, the flexural strength of the samples treated with 5A was evidently larger than that under the small current, and all the treated samples displayed enhanced flexural strength compared to that of untreated samples. The results depicted that the low-current treatment and short time could improve the interfacial properties between CF/epoxy, along with enhancing the flexural properties of the samples. However, a large amount of the joule heating from the larger current and a more extensive time frame is predicted to cause irreversible defects to the composite, which consequently leads to the reduction in flexural strength of the composite. TGA results indicated decreased thermal stability of the CFRE composite panels after the electric heating treatment was applied.

## 1. Introduction

Fibre-reinforced composite proves advantageous compared to other construction materials due to its properties, including lightweight, high tensile strength, higher strength–weight ratio, high corrosion resistance, cost-effectiveness, and convenient application because of its high flexibility. Due to its effective performance and cost, carbon fibre composites are increasingly applied in the transportation of infrastructure, aeroplanes, automobiles, and wind turbine blades [1,2,3,4,5]. It is widely recognised that land transportation is greatly affected by snow and ice climate disasters, and accidents or disasters may occur in serious cases. The carbon fibre composites are able to be utilised for strengthening and repairing building structures, along with melting ice and snow by further developing their own electric heating effect. Through this process the transformation of the work from passive maintenance to active maintenance can be promoted, thus reducing the harmful situations caused by snow and ice disasters in highway traffic. In relation to this, the de-icing behaviour of the carbon-fibre-reinforced polymer (CFRP) has been extensively studied recently [6,7,8]. Carbon fibre has consistently been used to prepare electrically heated concrete road plates, which can conveniently control the current parameters to de-ice and melt snow. As an electrically heated de-icing device, the internal temperature distribution of carbon fibre material contributes to its performance and has been systematically studied [5].

However, besides the advantage of the electrothermal effect, the research on material deterioration caused by electrothermal damage requires further analysis. A typical phenomenon occurring in the aeronautical sectors is lightning strikes, which have a current that is approximated to be in the range of 10~200 kA [9]. This can cause fibre rupture and fibre dissipation along several layers in the CFRP composite components, along with the resin vaporization near the point of discharge [10].

In addition, electric heating is also an effective method to prepare carbon fibre composites and improve their mechanical properties, such as impact, flexural, tensile, and fatigue properties [11,12,13,14,15]. The interfacial shear strength of carbon fibre in the composite sheet can also be improved, as well as its interfacial performance after the electrothermal heating treatment [11]. Previous studies [12,13] revealed that the impact resistance of carbon fibre composite can be improved by a short loading duration of a certain current, while a longer duration led to the loss of strength due to overpowering Joule heating effects. In a recent report, the CFRP composite laminates exposed to low values of DC current for 1–8 h led to a decreasing fatigue impact strength [15]. Therefore, more research on this is still needed before the wide application of the electrothermal effect in CFRE products.

The electrothermal effect originates from joule heating upon loading of an electric current on the samples. When the current is loaded onto CFRP laminates along the fiber axis, Joule heating takes place due to the resistive nature of carbon fibres, which has a general resistivity of 0.6–1.4 × 10^−3^ ohm/cm [16]. Under the lower currents, this Joule heating would only result in mild temperatures, which are expected to have some influence on the polymer matrix and the fiber/matrix interface. Extensive research has been implemented on the effects of temperature on the strength of CFRPs, which showed various effects on mechanical properties, especially in the transverse direction, and the damage mechanisms. It was revealed previously that the mode I fracture toughness (G_IC_) of the epoxy/carbon fiber laminated composites increased with the environmental temperature in the range of −80~50 °C [17]. Contrarily, the flexural properties deteriorated after a loading current of more than 8A, which led the temperature to increase to above 110 °C [18].

The electrical-conduction-induced Joule heating of composite laminates is suggested to be dependent on the laminate configuration [19]. In terms of electrothermal effect, carbon fibre plays a decisive role in CFRE composites. Its distribution and structural form correlate to the conductivity network in the composite material, which determines the electric heating efficiency of the CFRE composite plate. Compared with the common laminated CFRE composites, the composites prepared by the pultrusion method lack resin-rich interlayers. To some extent, this overcomes the weakness of the common continuous carbon fibre multi-layer composite laminates, which are prone to delamination damage and failure.

Taking into account these characteristics, the CFRE composite prepared with the pultrusion and moulding method has been used more widely in various structures, as well as those for multi-functions. For instance, the CFRP elements apply to both electrical heating and load-bearing in the structures, such as the de-icing devices in roads and bridges. However, it is noted that the majority of the research on the electrothermal effect of CFRP composites is focused on the laminates, providing minimal information on the electric heating behaviour of the CFRE prepared with the pultrusion method. In this work, the electrothermal effect and its influence on the mechanical properties, electric conduction, and thermal stability of CFRE sheet prepared by the pultrusion method were studied. This was conducted through the utilisation of an infrared thermal imager, thermogravimetric analysis, flexural performance test, and scanning electron microscope, respectively.

## 2. Experimental Materials and Instruments

### 2.1. Experimental Materials

**Figure 1:**
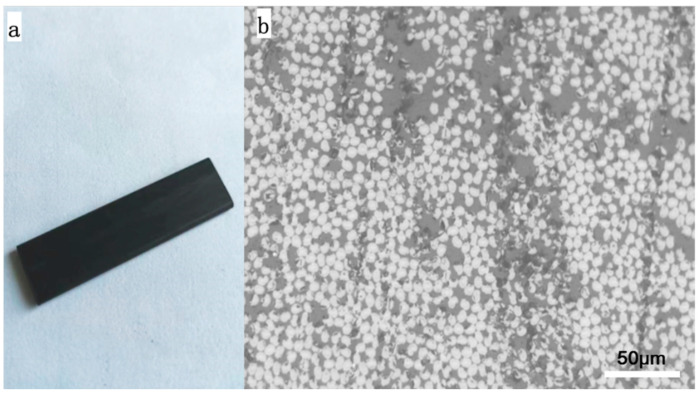
(**a**) The digital image of CFRE plate, and (**b**) the optical image of its cross-section.

The CFRE composite plates were fabricated and kindly supplied by Yangzhou Farber Carbon Fiber Products Co., Ltd., Yangzhou, China. The carbon fibre used in this test was T300, and the continuous carbon-fibre-reinforced epoxy resin matrix composite material plate was processed by employing the pultrusion moulding process. The carbon-fibre-reinforced composite sheet sample had a width of 10 mm and a thickness of 2 mm, as can be seen in Figure 1a. The fibre volume fraction of the composite sample was measured by the characterisation of its cross-section. As shown in the optical image of its cross-section (Figure 1b), all the carbon fibres are aligned perpendicular to the cross-section and a fibre volume fraction of 54.5% was obtained based on this image. It is noted that most of the fibre cross-sections were identified to be irregular circles in the matrix, which could be a characteristic of T300 carbon fibres. No layered structure of the fibre mat and resin-rich interlayer area was observed, which is distinguished from that of CFRE laminated composites previously prepared with the hand-layered method [18,19,20,21].

### 2.2. Test Instrument and Method

A scanning electron microscope (SEM, ULTRA55) produced by ZEISS, Germany, was used to observe the fracture surface of the sample. CFRE samples were coated with a gold layer before being put into the SEM chamber to alleviate the charging phenomena. Thermogravimetric analysis (TGA) tests of the samples were performed under N_2_ protection using a TGA/DSC3+ thermogravimetric analyser (Mettler tolido International Co., Ltd., Zürich, Switzerland) at a heating rate of 20 °C/min.

**Figure 2:**
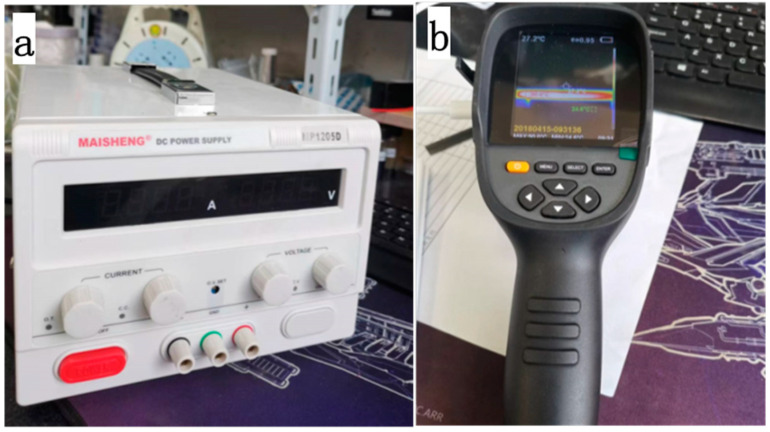
(**a**) The DC power supply, and (**b**) the infrared thermal imager.

**Figure 3:**
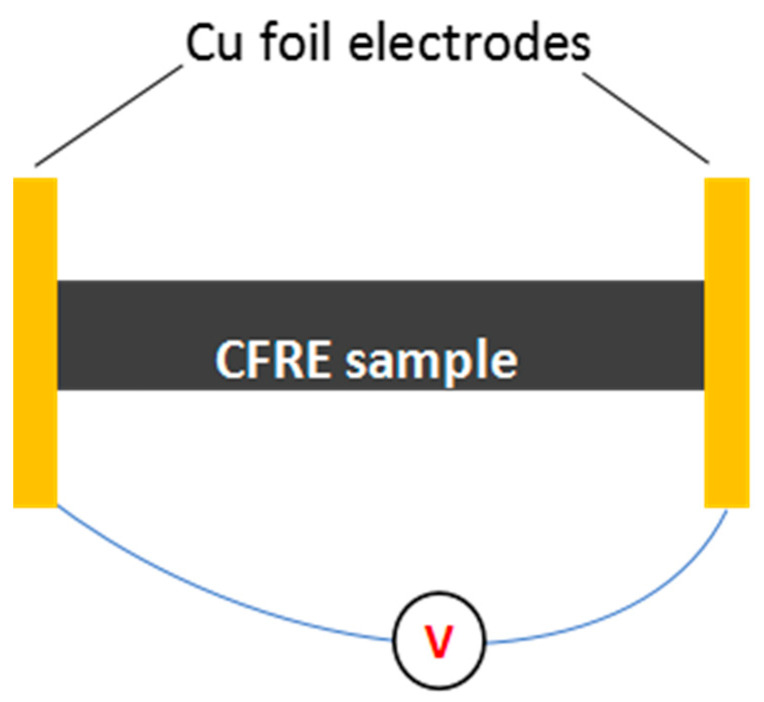
Wiring diagram for the electrothermal treatment of CFRE plate.

Masheng MP1205D high-precision digital display adjustable DC power supply (Figure 2a) was used to electrify the composite samples with a size of 40 mm × 10 mm × 2 mm. It had a voltage rating of 120 V and a current rating of 5A. The ambient temperature was 25 °C. Firstly, the sample was held between two copper electrodes and connected to the regulated power supply. Different DC currents were applied to the sample. During the current-loading process, the sample surface temperature was measured using the Xinst HT-19 infrared thermal imager without contact. The range of the measured temperature was −20–300 °C. The temperature distribution images of the CFRE sample surface under different conduction currents were taken, as shown in Figure 2b. To achieve the electrical heating of the samples, Cu foils were utilised as the electrode probes and attached to both cross-section side faces of the CFRE plate. The circuit for the current loading onto the samples was depicted in Figure 3.

According to ASTM/D7264 international test standard, the WDT mechanical test system of Shenzhen Kaiqiangli Test Instrument Co., Ltd., Shenzhen, China. was used to conduct bending mechanical tests on CFRE thin-plate samples subjected to different conduction currents. Specimens with the size of 80 mm (length) × 10 mm (width) × 2 mm (thickness) were cut with their length along the fibre’s longitudinal direction in the epoxy matrix. The flexural strength was calculated as follows:

(1) $$\sigma =\frac{3PL}{2bh^{2}}$$

where *σ*—the flexural strength of CFRE plate (MPa); *P*—maximum flexural load of CFRE plate (N); *L*—span (mm); *b*—CFRE plate width (mm); *h*—thickness of CFRE plate (mm).

The crosshead speed was set to be 2mm·min^−1^ and the span supporting the specimen was 64 mm for the three-point bending test.

## 3. Results and Discussion

### 3.1. Electrothermal Behavior of CFRE

**Figure 4:**
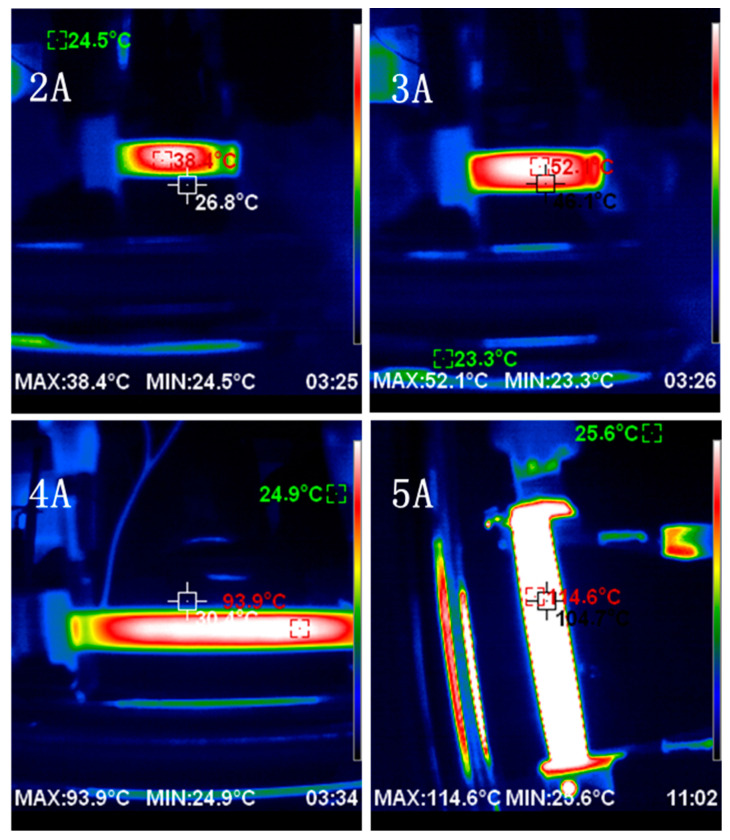
Infrared thermal imaging of CFRE plates with different conduction currents.

An infrared thermal imager was used to photograph the evolution of the surface temperature of CFRE sheet samples under different conduction currents (2~5A). Figure 4 portrays the infrared thermal imaging of the sample surface temperature when it reaches a relatively stable state after the loading of different currents. As can be seen from Figure 4, the temperature on the surface of the CFRE sample has a certain gradient distribution under the electrothermal effect. The temperature in the centre area of the CFRE thin-plate sample was higher, while the temperature at its edge was lower, in comparison. As shown in Figure 4, when the conduction current was 3A, the temperature of the white area in the centre of the sample surface reached 52.1 °C, while that of the red area near the edge was 46.1 °C. This was mainly due to the size effect of the CFRE sheet. As the sample temperature rise was derived from the electrothermal effect of the carbon fibre, which produced Joule heat, the accumulation of the Joule heat in the central regions of the sample surface resulted in a rapid temperature increase. Furthermore, it was observed that the closer the sample edge, which is affected more by environmental temperature, the greater the degree of heat dissipation, resulting in the presence of a certain temperature gradient. In addition, as shown in the image of the sample under 2A and 4A, it can be found that the thermal irradiation also led to higher environmental temperatures around the CFRE sample with larger current loading.

**Figure 5:**
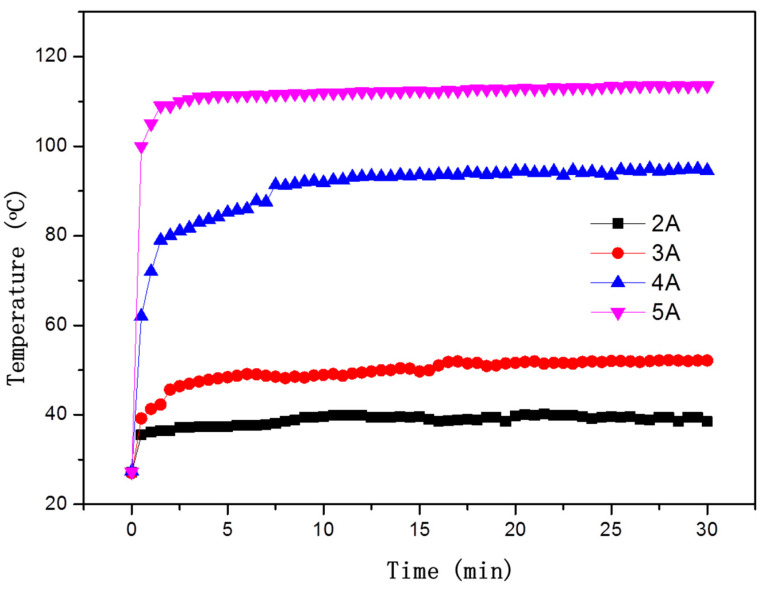
The relationship between surface temperature and time of CFRE plate under different conduction currents.

As shown in Figure 5, under four different conduction currents, the temperature of the CFRE samples rose rapidly at the beginning and reached an equilibrium state within approximately 2 min, and then remained generally stable with the time extension. By testing and comparing the temperature response behaviour of the CFRE samples under the loading of different conduction currents, it can be found that as the conduction current increased, the equilibrium temperature of the CFRE plate increased accordingly. According to Joule’s law, thermal energy generated by the electrothermal effect is proportional to the loading current, I^2^, for a certain resistance. When the current was 2A and 5A, the stable surface temperatures of the CFRE samples were 38.4 °C and 114.6 °C, respectively. This behaviour agrees with a similar research study [18]; however, the composite plate in this work was laminates and the current was much larger (≥ 8A). This could be attributed to the relatively uniform distribution of the carbon fibres in the polymer matrix in the pultruded CFRE composites of this work. The fast heating behaviour and high temperature at lower current loading proved beneficial for their functional applications, such as de-icing [5,6,7,8].

**Figure 6:**
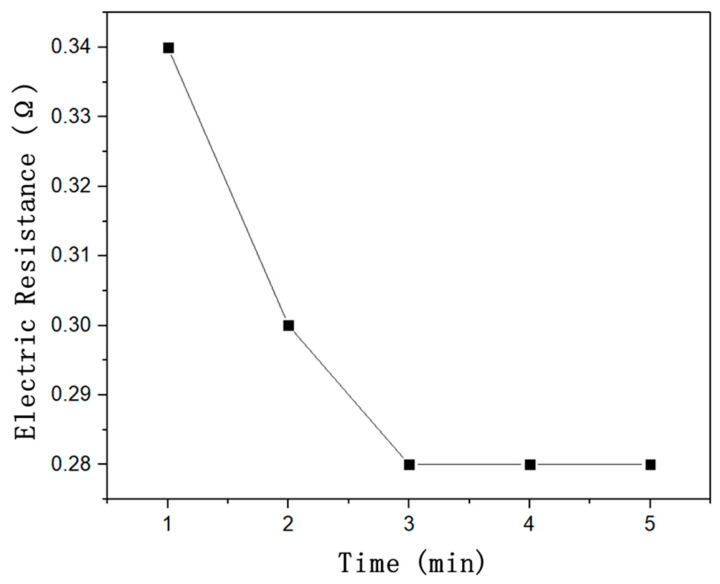
The relationship between the resistance of CFRE plate and time.

In addition, by monitoring the output voltage during the power-on process, the corresponding resistance of the sample with the power-on current was calculated. Figure 6 shows the dependence of the resistance of the CFRE thin-plate sample on current conduction time when the conduction current was 5A. As can be seen from Figure 6, the resistance of the CFRE sample decreased from 0.34 Ω to 0.28 Ω. At the initial stage of current conduction, the resistivity of the sample decreased rapidly due to the rapid rise of the sample temperature. After that, when the sample temperature increased slowly until equilibrium was reached, the sample resistance decreased gradually and gained stability. This could be due to the correlation between the conductivity of carbon fibre and its temperature. During the process of electrification, the increase of the internal temperature of carbon fibre due to the electrothermal effect led to the increased conductivity of the carbon fibre itself, therefore resulting in the resistance decrease of the CFRE plate.

### 3.2. Influence of Electrothermal Treatment on Flexural Performance of CFRE Plate

**Figure 7:**
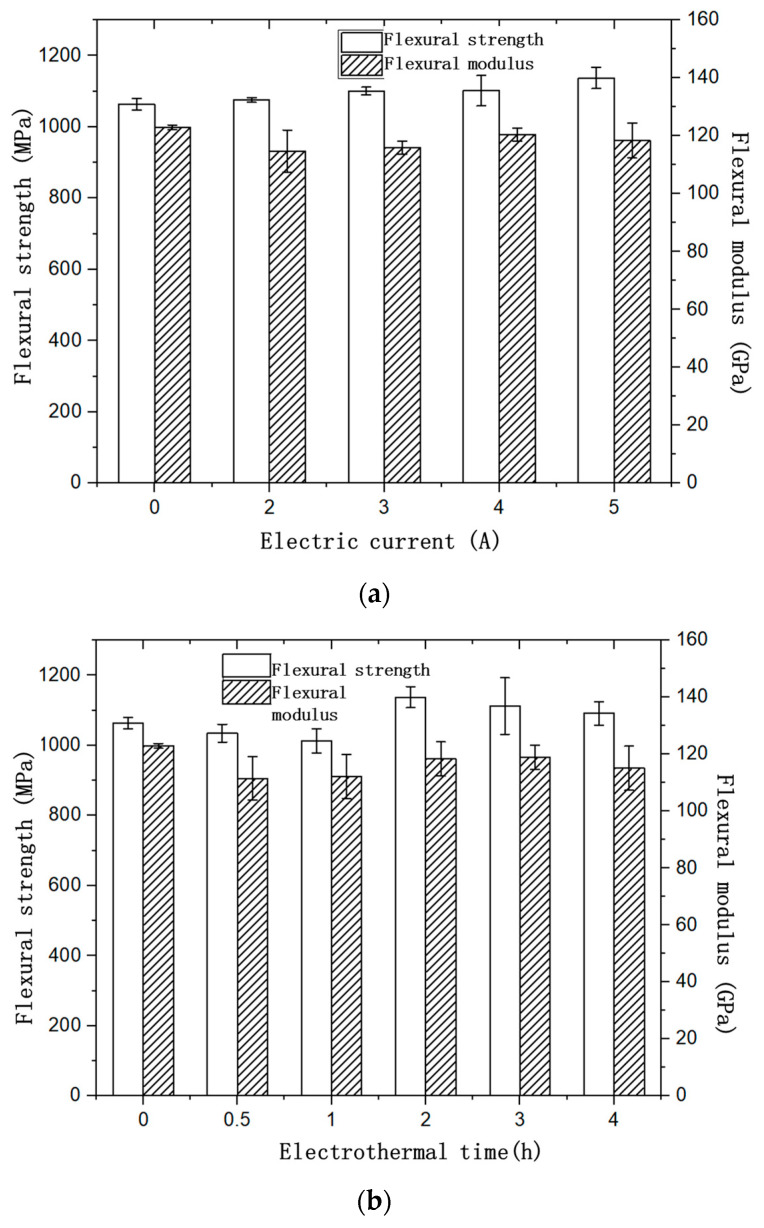
The relationship between the flexural performance of CFRE plate and conduction time and current. (**a**) The relationship between the conduction time and the flexural strength and modulus of the material with the current loading time of 2 h. (**b**) The relationship between the flexural strength and modulus of the material and the conduction current under 5A.

Based on the electrothermal effect of the CFRE composites, it was expected that a change in the mechanical properties of the samples would be observed. By undertaking the bending test, it was found that both the conduction time and the current have a great influence on the mechanical properties of CFRE composites. As shown in Figure 7a, when the current loading time was 2 h, the flexural strength of the CFRE sample after electrothermal treatment increased with the increase of the current. When the conduction current was 5A, the flexural strength reached the maximum value. Compared to the original CFRE composite plate, the flexural strength of CFRE composite samples treated with conduction current could be enhanced. The change in current loading time also affected the flexural strength of the CFRE samples. As represented in Figure 7b, when the energised current was set to be 5A, both the flexural modulus and flexural strength of CFRE composite material decreased first, and then increased with the increase of energised time. When the energised time was 2 h, the flexural strength reached the maximum value, and the flexural strength of the CFRE plate decreased with further loading time. The maximum flexural strength of the CFRE obtained via the electrothermal treatment under 5A for 2 h was about 7% larger than that without the treatment (0A). This enhancement was obviously bigger than that of CFRE laminate (0%) under 3A [22]. The statistical analysis of the flexural test results was processed with Microsoft EXCEL software, where both the standard deviation and *p*-values could be obtained. The standard deviation for the CFRE samples was obtained with the STDEVP function and presented as the corresponding error bar in Figure 7. It was found that the errors for all the flexural modulus data of the electrothermal treated samples in both Figure 7a,b were much larger than that of the sample without the treatment (0A). For the flexural strength data, the errors for the electrothermal treated samples in Figure 7 were comparable to that for the untreated sample, and some errors in Figure 7a were even less. Therefore, the *p*-values of the data were also obtained. As expected, the *p*-value for the flexural strength data in Figure 7a was 0.0092, which was less than that (0.0565) for the flexural strength data in Figure 7b. In contrast, the *p*-value for the flexural modulus data in Figure 7a,b was 0.0813 (Figure 7a) and 0.3334 (Figure 7b). These values were obviously larger than those for the corresponding flexural strength data. The statistical analysis result confirmed the reliability of the flexural strength data in comparison with the flexural modulus data in this work, and identified the significant effect of electrothermal time on the flexural strength of the CFRE composite. In consideration of the application environment of CFRP in various building fields [22,23,24,25,26], the improved flexural strength due to the electrothermal effect will make a difference in broadening their multi-function application.

It was suggested that the flexural strength of the CFRE composites was related to the interface bonding performance of carbon fibre/epoxy resin [11,18]. Therefore, by comparing the changes in the flexural strength of the samples before and after the loading of electric current, it was concluded that appropriate electrothermal treatment can improve the fibre/polymer interface, to a certain extent. This is conducive to improving the fibre/polymer interface and resulted in the increase of flexural strength of the CFRE composite. However, when the current increased to 5A along with exorbitant loading time, excessive Joule heat was generated in the CFRE sample. As carbon fibre was the heating source in the composites, irreversible damage to the interface between the fibre and epoxy matrix was caused, resulting in the deterioration of both the interface properties and the flexural properties of the sample.

### 3.3. Fracture Surface Morphology of CFRE Plate

**Figure 8:**
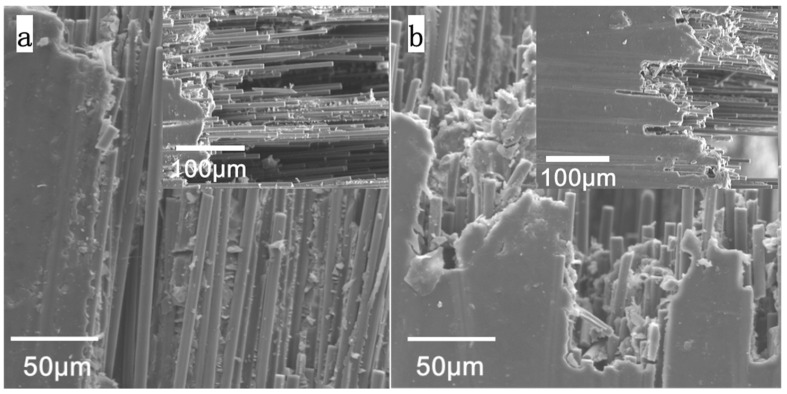
SEM images of fracture surfaces of CFRE samples. (**a**) Original sample, and (**b**) the sample after electrothermal treatment.

SEM was used to observe the morphology of the bending fracture surface of samples, further analysing the fracture mechanism of untreated and electrothermally heated samples. Figure 8a demonstrated the SEM photo of the untreated CFRE sample. It can be seen that a large number of long fibres in the untreated sample were pulled out from the matrix or exposed. Figure 8b shows the CFRE sample treated with the loading of 5A electric current for 2 h. The carbon fibres were pulled out on the fracture surface and their exposed length was much less than those in Figure 8a. Although more work needs to be done, the longer pull-out length implies that the 5A-treated CFRE composites have a better carbon fibre/epoxy interface, according to the previous research on the interfacial shear strength of the carbon fibre in the polymer matrix [27]. When the external force load was applied to the CFRE sample, the load was efficiently transferred to the fibre by the resin matrix. Once the external force load reached the critical value of composite fracture, the fibre either broke along with the resin matrix, or was partially pulled out. Therefore, it was proved that electrothermal treatment provided the CFRE composite with greater effectiveness regarding fibre interface and flexural strength.

### 3.4. Thermal Stability Analysis

**Figure 9:**
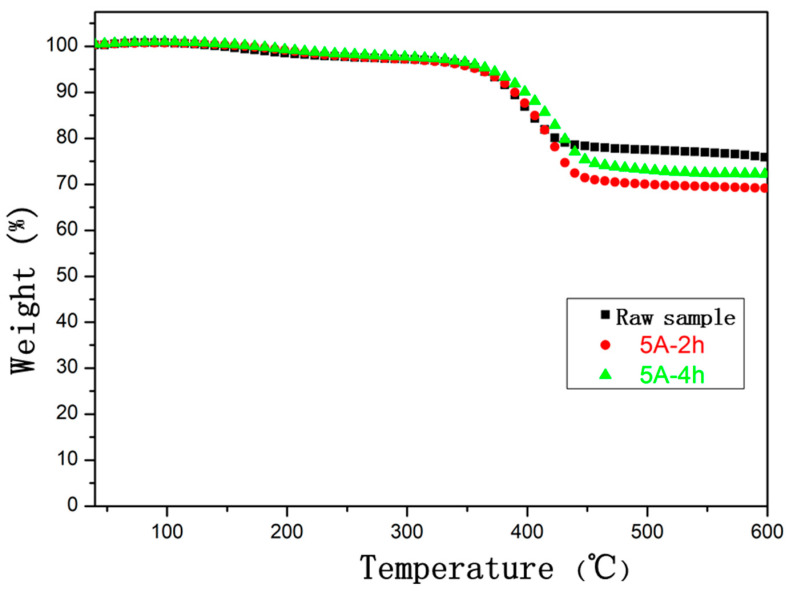
TGA curves of CFRE composites before and after electrothermal treatment.

Figure 9 displayed the thermogravimetry analysis curve of the CFRE composite plate samples before and after the electrothermal treatment. All three samples demonstrated a significant thermal weight loss at approximately 400 °C, which represented the thermal decomposition of epoxy resin matrix in CFRE composites. By comparing the TGA curves of the samples before and after electrothermal treatment, it can be found that the mass loss of all three CFRE samples was particularly different after 450 °C, and the difference was relatively stable with the temperature. At 500 °C, compared with the original sample, the weight difference of the composite caused by the electrothermal treatment with 5A for 2 h was 7.56%, while when the electrothermal treatment time was extended to 4 h, the weight difference decreased to 4.44%. This could be attributed to the different extent of the thermal decomposition of epoxy resin in CFRE composites originating from the different Joule heat generated under various electrothermal conditions. A shorter period of electrothermal treatment resulted in partial cracking and disconnection of polymer molecular chains and the decrease of molecular weight. Further, the Joule heat accumulated by an extensive duration of electrothermal treatment caused the pre-loss of thermally unstable small molecules in the composite material, which left more stable macromolecular chains of epoxy resin, leading to a rise in the thermal stability of the composites.

### 3.5. Electrothermal Response of Saturated Hygroscopic CFRE Plates

**Figure 10:**
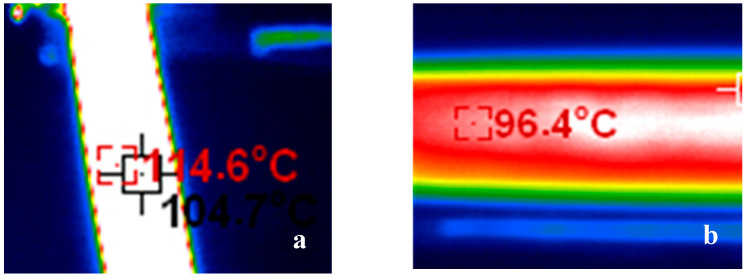
Infrared thermal imaging of CFRE plate before and after saturated hygroscopicity. (**a**) Original sample, and (**b**) saturated hygroscopic sample.

CFRE composite boards are being widely used in different humidity environments. In such cases, its electrothermal responses can be predictably affected by its absorption of water. Therefore, a preliminary study on the electrothermal response behaviour of CFRE with saturated hygroscopicity was conducted. The saturated hygroscopicity state of CFRE plates was identified by keeping it immersed in deionised water within a plastic box, which had a controlled temperature of 70 °C, based on the HB7401-1996 standard test [28]. Figure 10a,b elucidates the infrared thermal imaging of the surface temperature of the original CFRE sample and that with saturated hygroscopicity under the loading of the 5A conduction current, respectively. According to the standard, the saturated moisture absorption rate of CFRE material was tested to be 1.03% [28], which was obtained after its immersion in warm water for 3 days. Compared with the original sample without the hygroscopic treatment, the surface equilibrium temperature of the sample with the saturated hygroscopicity, which was 96.4 °C, was considerably lower than that of the original sample (114.6 °C). This was due to the fact that certain Joule heat needed to be consumed to remove the absorbed water in the epoxy resin matrix in CFRE composite during the electrothermal treatment process. The polymer chain segment in the composites, especially the polymer at the fibre/epoxy resin interface, caused a re-occurrence of the thermal cross-linking reaction. In addition, the evaporation of absorbed water caused defects in the CFRE, weakening the interfacial properties of the carbon fibres and reducing the thermal conductivity of CFRE materials as a whole. This led to a significant reduction in the surface equilibrium temperature of the CFRE composites. These results indicate that the water absorption of the CFRE composite had some negative effect on its electrothermal heating effect and should be considered in their design and application.

## 4. Conclusions

In this paper, the surface temperature response of CFRE composite prepared by the pultrusion process under different DC conduction currents and its influence on the mechanical properties of the sheet were studied. The resistivity, flexural property, and thermal stability of the composite were obtained. Additionally, the morphology of its fracture surface was observed, in order to analyse the influence of the electrothermal treatment on the bending fracture mechanism of CFRE composites. The conclusions are as follows:

1. Under the electrothermal effect, the higher the current, the higher the steady-state equilibrium temperature of CFRE samples. The CFRE sample could approach the steady-state equilibrium temperature within 2 min under 5A current.
2. The flexural performance of the CFRE composite was clearly affected by both the conducted current and time. The optimum electrothermal treatment conditions for improving material mechanical properties of the CFRE composite included a current loading of 5A for 2 h.
3. It was found that the appropriate electrothermal treatment could improve the carbon fibre/polymer interface in the CFRE sample.
4. Thermogravimetric analysis demonstrated that the thermal stability of CFRE composites decreased clearly after the electrothermal treatment.
5. Under the same electrothermal action, the achieved steady-state equilibrium temperature of the CFRE plate decreased significantly with the saturated hygroscopicity.

## Data Availability

Not applicable.
